# Detection and phylogenetic analysis of *Streptobacillus moniliformis*, the causative agent of rat-bite fever and Haverhill fever, in free-living greater bandicoot rats in Northeastern India

**DOI:** 10.14202/vetworld.2025.455-460

**Published:** 2025-02-19

**Authors:** A. A. P. Milton, Aleimo G. Momin, K. Srinivas, G. Bhuvana Priya, P. N. Gandhale, D. M. Firake, Samir Das, S. Ghatak, A. Sen

**Affiliations:** 1 ICAR Research Complex for NEH Region, Umiam, Meghalaya, India; 2 College of Agriculture, CAU (Imphal), Kyrdemkulai, Meghalaya, India; 3 ICAR-National Institute of High Security Animal Diseases, Bhopal, Madhya Pradesh, India; 4 ICAR-Directorate of Floricultural Research, Pune, Maharashtra, India

**Keywords:** bandicoots, Haverhill fever, India, rat-bite fever, *Streptobacillus moniliformis*

## Abstract

**Background and Aim:**

*Streptobacillus moniliformis* is the causative agent of zoonotic diseases such as rat-bite fever (RBF) and Haverhill fever (HF). While human infections are well-documented, limited studies have explored its presence in rodents in India. This study aimed to detect *S. moniliformis* in free-living bandicoots (*Bandicota bengalensis* and *Bandicota indica*) in Northeastern India and perform a phylogenetic analysis to assess its genetic relationship with global isolates.

**Materials and Methods:**

A total of 106 bandicoots (*B. bengalensis*, n = 76; *B. indica*, n = 30) were captured from various environments in Meghalaya, India. Fecal and tissue samples were collected and subjected to DNA extraction. Molecular detection of *S. moniliformis* was conducted using species-specific polymerase chain reaction (PCR) targeting the 16S ribosomal RNA gene. Positive amplicons were sequenced, analyzed using Basic Local Alignment Search Tool, and subjected to phylogenetic analysis.

**Results:**

PCR-based detection revealed a fecal prevalence of 3.3% (1/30) in *B. indica* and 0% in *B. bengalensis*. No tissue samples tested positive for *S. moniliformis*. The detected isolate exhibited 100% sequence identity with previously reported *S. moniliformis* strains and 99.63% similarity to *Streptobacillus notomytis*. Phylogenetic analysis clustered the recovered isolate with human and rodent-derived *S. moniliformis* strains from multiple global regions, suggesting potential zoonotic transmission.

**Conclusion:**

This study presents the first molecular detection of *S. moniliformis* in bandicoots from India, highlighting its zoonotic potential. Given its transmission risks through rodent bites and excreta contamination, public health surveillance is essential. Clinicians should consider RBF and HF in patients presenting with prolonged fever, particularly in rodent-endemic areas.

## INTRODUCTION

*Streptobacillus moniliformis* is the primary causative bacterium of the zoonotic diseases rat-bite fever (RBF) and Haverhill fever (HF) [1]. RBF was first reported in 1839 in the United States (US), and the association of *Streptothrix muris ratti* as the etiological agent was confirmed in 1916. The species was renamed *S. moniliformis* in 1925 [1]. An outbreak of *S. moniliformis* linked to milk consumption occurred in Haverhill, US, which was synonymous with HF [2]. Within the genus *Streptobacillus*, four other species have recently been described: *Streptobacillus ratti*, *Streptobacillus felis*, *Streptobacillus notomytis*, and *Streptobacillus canis* [3]. RBF has been attributed to both *S. moniliformis* and *S. notomytis*, whereas HF, a foodborne disease, is caused explicitly by *S. moniliformis* alone [4, 5]. *S. moniliformis* is a Gram-negative, pleomorphic, non-motile, and rod-shaped bacterium that is extremely fastidious, requiring microaerobic conditions for growth, which poses challenges for microbiological diagnosis. It exists in two forms: pathogenic bacillary and non-pathogenic cell wall-deficient L forms. Natural conversion between these two forms has been reported *in vitro*, and this phenomenon is speculated to contribute to resistance to treatment and clinical relapses [1, 6]. Human clinical cases of *S. moniliformis* are predominantly reported in the US and other American countries, such as Mexico, Canada, Brazil, and Paraguay. In Europe, cases have originated in the United Kingdom, Norway, France, Germany, Spain, Denmark, and other countries [1]. Sporadic reports are also available from Australia, Africa, and Asian countries, including China, Japan, and India [7–11]. RBF and HF are distinct clinical syndromes associated with *S. moniliformis* infection. The characteristic symptoms of RBF include fever, rigor, rashes, and migratory polyarthralgia. Conversely, the absence of rat exposure and the clustering of patients with HF at a specific time and place suggest the presence of HF, as the pathogen is typically transmitted to humans through the ingestion of milk, water, or food contaminated by rodent excretions [1, 4, 12]. A second outbreak of HF occurred in the United Kingdom in 1983, affecting 208 children at a residential school, and the outbreak was attributed to raw milk consumption [13, 14]. Penicillin is the antibiotic of choice for RBF and HF. The mortality rate in untreated cases is approximately 10%, with fatalities reported due to endocarditis, bronchopneumonia, refractory pericardial effusion, pneumonitis, volvulus, periarteritis nodosa, and overwhelming septicemia; endocarditis accounts for the majority of these deaths. Patients with HF exhibit symptoms similar to those of RBF [1]. Patients with *S. moniliformis*-associated endocarditis should receive combinatorial antibiotic therapy consisting of high-dose penicillin G and either gentamicin or streptomycin [1]. Rats serve as a natural reservoir for *S. moniliformis*, asymptomatically carrying the organism in their nasopharynx, upper trachea, larynx, and middle ear; symptoms of infection in rats have been occasionally observed [1]. Laboratory mice have also been shown to harbor pathogens that raise occupational health concerns for laboratory personnel [15]. Other animals, such as dogs, cats, guinea pigs, and ferrets, have been reported to become infected or colonized by *S. moniliformis*. However, confirmatory evidence regarding the transmission risk from dogs and cats to humans is lacking, as colonization typically occurs transiently after the consumption of rodents [1, 16].

There have been three clinical case reports of *S. moniliformis* in India, indicating the presence of the pathogen [9–11]. However, there are no documented cases of *S. moniliformis* being detected in rodents in India. Therefore, we initiated screening for *S. moniliformis* in free-living bandicoots that predominantly inhabit Meghalaya, a hilly state in Northeastern India.

This study aimed to detect *S. moniliformis* in blood, fecal, and tissue samples collected from rodents using polymerase chain reaction (PCR), followed by phylogenetic analysis to assess genetic similarity.

## MATERIALS AND METHODS

### Ethical approval

All procedures for handling rodents, including euthanasia and sample collection, were approved by the Institutional Animal Ethics Committee of the Institute and were registered with the Committee for Control and Supervision of Experiments on Animals (V-1101(13)/122023-CPCSEA-DADF).

### Study period and location

From January 2020 to March 2022, free-living bandicoots, specifically *Bandicota indica* (Greater Bandicoot Rat) and *Bandicota bengalensis* (Lesser Bandicoot Rat), were captured in the Ri-Bhoi and East Khasi Hills districts of Meghalaya (25.4670° N, 91.3662° E), a hilly state in Northeastern India.

### The capture of bandicoots

Synanthropic bandicoots were caught using Sherman and indigenous traps set in various locations, including houses, restaurants, vegetable markets, wet food markets, animal feed storage rooms, livestock farms, and agricultural fields. In total, 106 bandicoots were captured, including 76 *B. bengalensis* and 30 *B. indica*. The sample size was based on systematic trapping across multiple locations over a defined period, given the unpredictability of free-ranging wildlife. All captured rodents were included without bias and appeared healthy at the time of trapping.

### Fecal and tissue samples

The bandicoots were euthanized using chloroform, followed by cervical dislocation. Fecal materials were collected from the intestines, and tissue samples (liver, spleen, lungs, kidney, and brain) were pooled and labeled. All samples were stored at −20°C until further analysis.

### DNA extraction

Genomic DNA from fecal samples was extracted using the QIAamp PowerFecal Pro DNA Kit (Qiagen, Hilden, Germany), whereas DNA from pooled tissue samples was extracted using the DNeasy Blood and Tissue Kit (Qiagen). The extraction procedures were performed according to the manufacturer’s instructions.

### PCR analysis of *S. moniliformis*

All genomic DNAs extracted from fecal and tissue samples were subjected to PCR screening for detecting *Streptobacillus moniliformis*. Following initial screening, positive amplicons were sequenced for confirmation and used as positive controls. Subsequently, all samples were re-screened using a standardized PCR protocol. We used the PCR protocol targeting the *16S ribosomal RN*A (rRNA) gene of *S. moniliformis*, as developed by Kimura *et al*. [12]. The primer sequences used were S5F- 5′-CATACTCGGAATAAGATGG-3′ and AS2R- 5′-GCTTAGCTCCTCTTTGTAC-3′, yielding an amplicon size of 269 bp. PCR was conducted in a Mastercycler nexus GX2 (Eppendorf, Germany) with a 25 μl reaction mixture containing 12.5 μL of 2× Dream Taq Master Mix (Thermo Fisher Scientific, Waltham, MA, US), 1 μL (10 pmol) of each primer, and 2.5 μL of the test DNA. The PCR program included an initial denaturation step at 95°C for 3 min, followed by 35 cycles of denaturation at 95°C for 20 s, annealing at 57°C for 1 min, extension at 72°C for 1 min, and a final extension at 72°C for 7 min. PCR amplicons were electrophoresed on a 2.5% agarose gel and documented using a gel imaging system (Vilber Lourmat, Marne-la-Vallée, France).

### Sequencing and phylogenetic analysis

Positive PCR amplicons were sequenced using the Sanger method, and sequencing was outsourced to Eurofins Genomics India Pvt. Ltd., Bengaluru, India. Quality checks and sequence assembly were performed using MEGA 11 [17]. Taxonomic identification of the assembled sequences was conducted using the National Center for Biotechnology Information (NCBI) Basic Local Alignment Search Tool (BLAST). The sequences were subsequently submitted to GenBank for confirmation. A phylogenetic tree was constructed, including 19 previously submitted nucleotide sequences from around the world, using the maximum likelihood algorithm with MEGA 11. The Jukes-Cantor Model was selected based on the Bayesian Information Criterion = 1343.586. The tree with the highest log likelihood (−493.63) is presented, indicating the percentage of trees with associated taxa clustered together next to the branches. This analysis involved 20 nucleotide sequences comprising 267 positions in the final dataset, and evolutionary analyses were conducted using MEGA 11.

## RESULTS

### Identification of *S. moniliformis*

**Figure 1:**
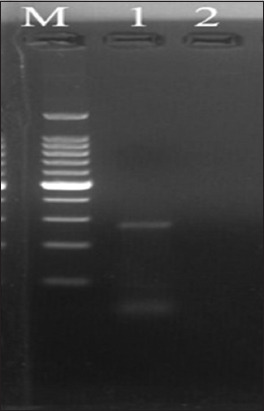
Polymerase chain reaction amplification of the 16S ribosomal RNA gene of *Streptobacillus moniliformis* showing the desired positive amplicon of 269 bp (M: 100 bp plus DNA marker, Lane 1: Positive sample; Lane 2: Non-template control).

Molecular screening using PCR targeting the 16S rRNA gene of *S. moniliformis* revealed a fecal prevalence of 0.94% (1/106 samples) in bandicoots, whereas no *S. moniliformis* was detected in tissue samples (0%). The specific prevalence of *S. moniliformis* in *B. indica* and *B. bengalensis* was 3.33% (1/30) and 0% (0/76), respectively, yielding an expected amplicon size of 269 bp (Figure 1).

### Sequencing and phylogenetic analysis

**Figure 2:**
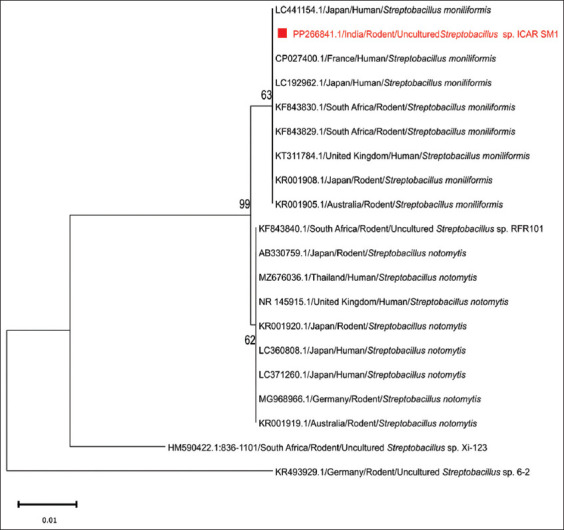
Phylogenetic analysis (PP266841.1 is the National Center for Biotechnology Information Accession Number obtained for the *Streptobacillus moniliformis* strain of the present study).

The positive amplicon (269 bp) was sequenced in both directions, assembled, and submitted to NCBI, resulting in the acquisition of the accession number (PP266841.1). BLAST analysis confirmed that the detected isolate was *S. moniliformis*, with a 100% identity match to previously submitted sequences of this species (Table 1). The sequences from the current isolate shared 99.63% identity with the sequences of *S. notomytis* (Table 1), indicating a close phylogenetic relationship between these species. For the phylogenetic analysis, we included rodent and human strains of *S. moniliformis* and *S. notomytis* from various regions, including the United Kingdom, France, Germany, Thailand, Japan, Australia, and South Africa. The resulting phylogenetic tree shows two main clusters representing *S. moniliformis* and *S. notomytis*. Our *S. moniliformis* strain recovered from *B. indica* clustered with *S. moniliformis* strains from human and rodent sources in other countries (Figure 2). This clustering pattern provides insights into the genetic similarity and evolutionary lineage of these *Streptobacillus* spp. across different hosts and geographical locations. Furthermore, this study highlights the zoonotic potential of *S. moniliformis*, as the clustering of strains from rodents and humans suggests a shared evolutionary history and the potential for cross-species transmission.

**Table 1:** Details of sequences included in the phylogenetic tree along with query coverage and percentage identity with the isolate recovered in the current study (PP266841.1).

Accession no.	Taxonomy	Isolation source	Country	Query coverage versus PP266841.1	Percentage identity with PP266841.1
KR001905.1	*S. moniliformis*	Rodent	Australia	100	100
KR001908.1	*S. moniliformis*	Rodent	Japan	100	100
KT311784.1	*S. moniliformis*	Human	United Kingdom	100	100
KF843829.1	*S. moniliformis*	Rodent	South Africa	100	100
KF843830.1	*S. moniliformis*	Rodent	South Africa	100	100
LC192962.1	*S. moniliformis*	Human	Japan	100	100
CP027400.1	*S. moniliformis*	Human	France	100	100
LC441154.1	*S. moniliformis*	Human	Japan	100	100
KF843840.1	Uncultured *Streptobacillus* spp*.*	Rodent	South Africa	100	99.63
AB330759.1	*S. notomytis*	Rodent	Japan	100	99.63
MZ676036.1	*S. notomytis*	Human	Thailand	100	99.63
NR_145915.1	*S. notomytis*	Human	United Kingdom	100	99.63
KR001919.1	*S. notomytis*	Rodent	Australia	100	99.63
KR001920.1	*S. notomytis*	Rodent	Japan	100	99.63
LC360808.1	*S. notomytis*	Human	Japan	100	99.63
LC371260.1	*S. notomytis*	Human	Japan	100	99.63
MG968966.1	*S. notomytis*	Rodent	Germany	100	99.63
HM590422.1	Uncultured *Streptobacillus* spp*.*	Rodent	South Africa	99	95.11
KR493929.1	Uncultured *Streptobacillus* spp*.*	Rodent	Germany	94	92.86

*S. moniliformis*=*Streptobacillus moniliformis, S. notomytis*=*Streptobacillus notomytis*

## DISCUSSION

*S. moniliformis*, an important zoonotic bacterial pathogen known to cause RBF and HF, has been isolated, with numerous human clinical cases reported worldwide [1]. However, this pathogen has never been reported in *B. indica*, and there are no existing reports of *S. moniliformis* in rodents in India. This study represents the first report of *S. moniliformis* in Indian bandicoots, with a fecal prevalence of 3.3% in *B. indica*, which holds significant implications from zoonotic and public health perspectives. There are clinical reports of *S. moniliformis* infection in humans in India. The first case involved an 18-year-old male patient with congenital heart disease who developed endocarditis caused by *S. moniliformis*. The organism was isolated through blood culture, and the suspected source of infection was consumption of water or food contaminated with rodent feces. The patient exhibited clinical signs, such as fever, cough, joint pain, and epistaxis, and responded well to treatment with penicillin and gentamicin [9]. In a second report by De *et al*. [10], *S. moniliformis* was isolated from the blood culture of a child with acute lymphoblastic leukemia. Similar to the first case, there was no history of a rat-bite, and the source of infection was believed to be food or water contaminated with rat excreta. The child presented with fever, diarrhea, vomiting, and purpuric spots on the lower extremities and showed improvement after treatment with cefotaxime and amikacin [10]. Third report from India [11] involved a 44-year-old man with mitral valve endocarditis, from whom *S. moniliformis* was isolated through blood culture. The patient had a history of being bitten by an unknown small creature and responded positively to treatment with penicillin and gentamicin treatment [11]. These clinical cases of *S. moniliformis* in humans highlight the presence of this pathogen in India and highlight the potential zoonotic risk associated with rodent interactions.

In this study, we determined the fecal prevalence of *S. moniliformis* in Indian bandicoots using PCR-based analysis. While human infections are typically confirmed by culturing blood, cerebrospinal fluid, or joint aspirates, the slow growth of this bacterium hampers rapid confirmation [18]. Previous studies by Balakrishnan *et al*. [3], Kimura *et al*. [12], Boot *et al*. [19] and Andre *et al*. [20] have successfully used PCR-based detection of *S. moniliformis* in samples from both patients and rodents. The PCR protocol employed in our study was developed by Kimura *et al*. [12], who targeted the 16S rRNA gene to specifically detect *S. moniliformis*. Kimura *et al*. [12] noted that the PCR assay developed by Andre *et al*. [20] requires post-amplification nucleotide sequencing due to the use of universa*l* 16S rRNA primers. In contrast, the protocol described by Boot *et al*. [19] showed non-specific amplification. Thus, the PCR method developed by Kimura *et al*. [12] is more accurate and specific for direct screening of samples for *S. moniliformis*.

Given the recent identification of new species, such as *S. ratti*, *S. felis*, *S. notomytis*, and *S. canis*, we sequenced the positive PCR amplicons to confirm the presence of these species [3]. The isolate from this study was confirmed as *S. moniliformis*, matching 100% with previously submitted sequences of this species and sharing 99.63% similarity with *S. notomytis*. Phylogenetic analysis, which included strains from both rodent and human origins worldwide, indicated that the isolate recovered from *B. indica* is indeed *S. moniliformis*, with strains from rodent and human sources clustering together, suggesting the potential for zoonotic transmission globally.

Despite numerous clinical reports of *S. moniliformis* infection in humans, there is a notable lack of studies documenting the prevalence of this pathogen in various rodent species. For instance, a study conducted in Iran reported a fecal prevalence of 23% in wild Norway rats (*Rattus norvegicus*) [21]. Consistent with our findings, a study conducted in Iraq noted a low prevalence of 1.66% in the pharyngeal swabs of the black rat (*Rattus rattus*) [22]. In addition, a previous PCR-based screening of *Streptobacillus* spp. in *Rattus* spp. in South Africa reported prevalences of 50.94% and 1.61% in oral swabs and kidney samples, respectively [18]. Although the prevalence in our study was low, rodents can act as reservoirs for the organism, potentially excreting it through feces and urine. This can lead to contamination of food and water sources, facilitating the entry of *S. moniliformis* into the food chain and contributing to potential outbreaks. Therefore, surveillance studies across various regions are warranted to determine the rates of host colonization relative to geographical variations, which is crucial for informing public health authorities in each region.

## CONCLUSION

This study presents the first molecular detection of *S. moniliformis* in free-living bandicoots in India, with a fecal prevalence of 3.3% (*B. indica*) and no detection in *B. bengalensis*. Phylogenetic analysis confirmed a close relationship between the identified strain and previously reported *S. moniliformis* isolates from rodents and humans worldwide, indicating potential zoonotic transmission. These findings highlight the need for heightened public health awareness regarding rat-borne bacterial pathogens and their role in disease transmission.

This study provides novel evidence of *S. moniliformis* in Indian rodents, filling a critical gap in epidemiological data. The use of PCR and sequencing ensured specific detection, avoiding false positives, while a global phylogenetic comparison strengthened the evidence of zoonotic transmission potential. However, the study has limitations, including a moderate sample size restricted to a specific region, the lack of environmental and host factor analysis, and the absence of a direct linkage between infected rodents and human cases.

Future studies should focus on expanding surveillance to cover different geographic regions and rodent species to determine the broader epidemiology of *S. moniliformis* in India. Investigations into host-pathogen interactions, environmental factors, and antimicrobial resistance profiles could provide deeper insights into transmission dynamics. In addition, human serosurveys and case-based studies are necessary to assess the actual disease burden and public health significance of *S. moniliformis* infections. This study underscores the importance of rodent surveillance in zoonotic disease monitoring and highlights the need for integrated public health measures to mitigate potential risks associated with *S. moniliformis* transmission.

## AUTHORS’ CONTRIBUTIONS

AAPM: Conceptualized the study, investigation, data analysis, and drafted the manuscript. AGM: Investigation. KS: Analysis. GBP: Data analysis and drafted the manuscript. PNG: Conceptualized the study. DMF: Investigation (rodent identification). SD: Investigation and edited the manuscript. SG and AS: Data analysis and reviewed the manuscript. All authors have read and approved the final manuscript.
